# Subgingival Periopathogens Assessment and Clinical Periodontal Evaluation of Gastric Cancer Patients—A Cross Sectional Pilot Study

**DOI:** 10.3390/pathogens11030360

**Published:** 2022-03-16

**Authors:** Flavia Mirela Nicolae, Andreea Cristiana Didilescu, Petra Șurlin, Bogdan Silviu Ungureanu, Valeriu Marin Șurlin, Ștefan Pătrașcu, Sandu Ramboiu, Igor Jelihovschi, Luminita Smaranda Iancu, Mirela Ghilusi, Mihai Cucu, Dan Ionuț Gheonea

**Affiliations:** 1 Department of Periodontology, University of Medicine and Pharmacy of Craiova, 200349 Craiova, Romania; flavia.nicolae23@yahoo.com; 2 Department of Embryology, Faculty of Dental Medicine, Carol Davila University of Medicine and Pharmacy, 8 Eroii Sanitari Boulevard, 050474 Bucharest, Romania; andreea.didilescu@umfcd.ro; 3 Department of Gastroenterology, University of Medicine and Pharmacy of Craiova, 200349 Craiova, Romania; digheonea@gmail.com; 4 Department 1st of Surgery, University of Medicine and Pharmacy of Craiova, 200349 Craiova, Romania; vsurlin@gmail.com (V.M.Ș.); stef.patrascu@gmail.com (Ș.P.); sandu_r@yahoo.com (S.R.); 5 Department of Preventive Medicine and Interdisciplinarity, Grigore T. Popa University of Medicine and Pharmacy, 700115 Iași, Romania; jelihovschi.igor@umfiasi.ro (I.J.); luminita.iancu@umfiasi.ro (L.S.I.); 6 Department of Pathology, Clinical County Emergency Hospital of Craiova, 200349 Craiova, Romania; mirela@dass.ro; 7 Department of Genetics, University of Medicine and Pharmacy of Craiova, 200349 Craiova, Romania; mihai.cucu@geneticamedicala.ro

**Keywords:** *Fusobacterium nucleatum*, periodontal disease, gastric cancer, periopathogens

## Abstract

Oral microbiota have shown a higher bacterial diversity in patients with cancers of the digestive tract, with higher levels of periopathogens. Recent studies have shown that *Fusobacterium* links to gastro-intestinal neoplastic tissue and accelerates its progression, as well as worsening patient outcome. The present pilot study was carried out between February and December 2020 to evaluate the possible association between the abundance of some periopathogens (*Fusobacterium nucleatum*, *Porphyromonas gingivalis*, *Aggregatibacter actinomycetemcomitans*, *Treponema denticola* and *Tannerella forsythia*) in subgingival plaque and periodontal status with characteristics of gastric cancer. The study was performed on a sample of 24 patients with gastric cancer from the 1st Department of Surgery and Department of Gastroenterology within the Clinical County Hospital of Emergency of Craiova, Romania. The patients’ oral cavity was examined, gingival crevicular samples were collected, and signs of periodontal disease were recorded. On the histopathological exam, the differentiation grade and size of the tumour were registered. Our results showed that, from the periopathogens studied, the most abundant bacteria were *F. nucleatum* followed by *T. forsythia* in all groups. In our present study, the strong correlation between tumour dimension and all periodontal parameters but also between tumour dimension and *F. nucleatum* could suggest a positive association between periodontal disease, tumoral growth and periopathogens implication in this process.

## 1. Introduction

Periodontitis is a chronic inflammatory disease which leads to the destruction of the tooth-supporting tissues. Clinically, it translates into periodontal pockets, clinical attachment loss (CAL), bleeding on probing (BOP) and alveolar bone loss, and if left untreated, it can lead to tooth loss. Its main aetiology is microbiological, mainly bacteria from the subgingival biofilm, but it has many multifactorial aggravating factors, both local and systemic [1,2]. Among the risk factors for periodontitis are smoking, diabetes mellitus, psychological stress, genetic factors, host response, osteoporosis and ageing, some being modifiable, and some not [3]. 

It was estimated that more than 700 bacterial species reside in the oral cavity, and most of them are commensal bacteria, but in conditions of imbalances, some of the bacteria from the subgingival biofilm could become pathogens for the tooth-supporting structures, the periodontium. The subgingival biofilm was first classified by Socransky in colorimetrically coded complexes. The first three ones, yellow, green and purple, are found in periodontal health, mainly streptococcal species, *Capnocytophaga* species, *Eikenella corrodens*, *Actinomyces odontolyticus* and *Veillonella parvula*, whereas the orange one plays a key role in the progression of the disease, serving as a bridge between health status and disease. Without the bacteria from the orange complex (*Fusobacterium nucleatum*, *Prevotella intermedia*, *Campylobacter rectus*), the more aggressive bacteria from the last complex, the red one (*Porphyromonas gingivalis*, *Treponema denticola*, *Tanerella forsythia*), may not adhere [4].

*Fusobacterium* not only has an important role in the progression of periodontitis, but in the last few years, it has been suggested that it also plays a major role in systemic diseases. It has been linked with adverse pregnancy outcomes [5], atherosclerosis [6], cerebral aneurysm [7], Alzheimer’s disease [8], and gastro-intestinal disorders such as appendicitis [9], inflammatory bowel disease [10], colorectal [11] and gastric cancer (GC) [12]. More and more studies show that the bacterium links to the gastro-intestinal neoplastic tissue and accelerates its progression, as well as worsens patient outcome [13,14,15,16,17,18].

The oral microbiota showed a higher bacterial diversity in patients with cancers of the digestive tract, with higher levels of periopathogens *P. gingivalis*, *T. forsythia*, *T. denticola* and *Aggregatibacter actinomycetemcomitans* [19,20,21]. 

Gastric cancer (GC) is in fifth place among cancers regarding incidence, and it represents about 8.2% of all cancer deaths. There is an important variation in incidence and mortality between regions all over the globe, the highest incidence being reported in Asian countries such as Japan, China and South Korea. and the lowest reported in Northern America and Northern Europe, while in Romania, the GC incidence has still been quite high over recent years [22]. According to the scientific literature, the most prevalent forms of GC are adenocarcinomas, accounting for over 95% [23]. In the stomach, there is normally a resident microbiota, among which *Streptococcus*, *Proteobacteria*, *Bacteroides*, *Firmicutes* and *Actinobacteria* can be found. In addition, bacteria transiting from the oral cavity, such as *Veillonella*, *Fusobacterium* and *Prevotella*, have been discovered [24]. Numerous risk factors such as body mass index, smoking, low socioeconomic status, chronic inflammation of the gastric tissue, and *Helicobacter pylori* infection have been incriminated in the appearance and evolution of GC [25]. 

There are scientific articles, inhomogeneous in aim and methodology, regarding the relationship between periodontal disease (PD) and the periodontal microbiome, as well as regarding the characteristics of GC and the presence of periopathogens in gastric neoplastic tissues. After analysing the gastric microbiota, increased numbers of *F. nucleatum* and *Clostridium colicanis* were found in gastric neoplastic tissues, and it was stated that these bacteria have an oral origin, explaining a possible dissemination of the bacteria through an enteral route [12].

Other results found abnormal increases in saliva and dental plaque of two periopathogens on a group of patients with GC compared to control [26]. These papers did not investigate oral or periodontal status, while a case-control study highlighted that the severity of PD is more elevated in GC patients. The authors analysed the periodontal status of patients with GC, without an analysis of the periodontal microbiota or a more detailed description of gastric neoplastic tissue [27]. 

### Aim

The present pilot study was carried out to assess the possible association of the abundance of some subgingival periopathogens (*F. nucleatum*, *P. gingivalis*, *T. denticola*, *T. forsythia*, *A. actinomycetemcomitans*) and periodontal status regarding the characteristics of GC.

## 2. Results

### 2.1. Prevalence of Periopathogens 

In the gingivitis group (G—10 patients), *F. nucleatum* was present in the gingival crevicular fluid in all 10 cases, *A. actinomycetemcomitans* and fadA were not detected in any of the samples. *P. gingivalis*, *T. denticola* and *T. forsythia* were present in different percentages, *T. forsythia* being present in 9 of the 10 cases (90%) (Table 1).

In the periodontitis group (P—14 patients), *F. nucleatum* and *T. forsythia* were detected in all 14 cases, *fadA*, *P. gingivalis*, *A. actinomycetemcomitans* and *T. denticola* were present in different percentages but were higher than in group G. (Table 1)

**Figure 1:**
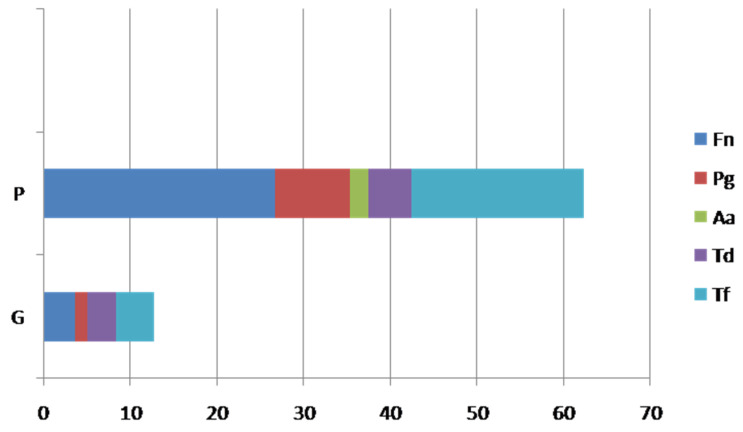
The subgingival abundance of studied periopathogens in patients with gastric cancer. Fn, *Fusobacterium nucleatum*; Pg, *Porphyromonas gingivalis*; Aa, *Aggregatibacter actinomycetemcomitans*; Td, *Treponema denticola*; Tf, *Tannerella forsythia*.

The abundance of bacteria in each group is represented in Figure 1, with a statistically significant difference (*p* < 0.05) between the G and P groups.

### 2.2. Correlations between Periodontal Status and Characteristics of Gastric Tumours

The values of probing pocket depth (PPD), CAL, and BOP, and the number of absent teeth (AT) in the G and P groups are presented in Table 1. 

In patients with periodontitis from the surgery department (SP) (*n* = 12), to whom it was possible to measure the tumour dimension, strong significant correlations were found between TD with BOP (r = 0.973, *p* = 0.00036), PPD (r = 0.963, *p* = 0.00055), AT (r = 0.957, *p* = 0.000024) and a strong significant correlation between TD with CAL (r = 0.684, *p* = 0.005).

The prevalence of differentiation grade (DG) in the three studied groups is presented in Table 2. In all groups, the most prevalent DG was the poor one.

No correlations were found between DG and the periodontal status of patients.

### 2.3. Correlations between Abundance of Subgingival Periopathogens in Gingival Crevicular Fluid and Characteristics of Gastric Tumours

A strong significant correlation was found between TD and *F. nucleatum* (r = 0.968, *p* = 0.00013), and a moderate significant correlation was found between TD and *T. forsythia* in the SP group (r = 0.413, *p =* 0.0053). 

No correlations were found between the abundance of periopathogens and DG in any of the studied groups. 

## 3. Discussion

Considering that the aim of our study was to evaluate a possible link between the clinical and microbiological periodontal parameters and the characteristics of GC, the correlations we found - strong correlation of TD with all the assessed periodontal parameters (AT, PPD, BOP, and CAL) in patients periodontally affected, as well as of TD with *F. nucleatum* and the moderate correlation of TD with *T. forsythia*, might suggest a positive association between periodontal disease, tumoral growth, and the periopathogens’ implication, especially for *F. nucleatum* in this process. Taking into account that this is a pilot study, with a limited number of patients, these results should be confirmed by further studies with a larger number of patients.

Regarding the number of absent teeth, our results showed that there were 4–8 teeth lost in GC patients with gingivitis and 6–15 in those with periodontitis diagnostic, in accordance with the scientific literature. A meta-analysis from 2016 hypothesized that tooth loss could be linked with the onset of GC [28], and Ndegwa et al. had considered that patients with tooth loss and denture-associated lesions were at a higher risk of GC [29]. 

Studies linking tooth loss to GC have been published since 1976, and it was believed that edentulism was a predictor of the disease [30]. In the 1990s, a study showed a two-fold increased risk of GC in patients who lost more than 10 teeth [31], which is similar with a study by Abnet et al., who reported a two-fold increase in the risk of GC in edentulous patients, in comparison with patients who lost 10 teeth [32]. In a recent study, with a 22- to 28-year follow-up, an increased risk of 52% for developing gastric adenocarcinoma was found in patients with a history of PD. In addition, the risk was more elevated in patients who lost more than two teeth [27]. Not only was GC associated with tooth loss, but other results also associated tooth loss with the occurrence of gastro-intestinal cancers [33,34,35,36,37,38].

The PPD that we measured in our study was greater or equal to 6 mm in 64.28% of the patients, and all the patients with periodontitis had at least one pocket ≥ 6 mm. Researchers have also found PPD greater or equal to 6 mm in 76% of the patients with oral or oropharyngeal cancer [39]. PPD and the number of remanent teeth of patients suffering from GC were found by Matsuda et al., similar with other patients suffering from colorectal and pancreatic cancer [40], but one research did not find any statistically significant differences regarding PPD of GC patients vs. controls [19].

The risk of developing cancer rose to 17% in patients suffering from PD, and it was found that in patients with moderate or severe periodontitis, the risk of neoplastic tissues is higher than in patients with milder forms, although their risk is not negligible [41]. In a recent review from 2020, Nwizu et al. pointed out a positive association between PD and cancer risk and hypothesized that, regarding PD, certain anatomic sites may be at higher risk for developing neoplasia, especially the esophagus and upper gastro-intestinal tract, which are adjacent to the oral cavity [42].

Another study provides epidemiological data on whether the history of PD is associated with a 53% increased risk of gastric adenocarcinoma, highlighting the importance of the oral microbiome in GC and recommending prospective studies to identify specific oral bacteria responsible for this association [43]. 

Boehm et al. [18] found similar levels of *F. nucleatum* in GC tumour tissues and the adjacent mucosa, but they were lower than in the tumorous and non-tumorous sites of colorectal cancers, and he assigned a bad prognosis to patients with GC, with a worse overall survival rate in patients with *F. nucleatum* attached to the tumorous tissue [18]. Considering the hypothesis that oral bacteria are swallowed and delivered to the lower intestinal tract, higher levels of fusobacteria from CRC could be explained [20], but regarding the relatively low levels from GC, Boehm’s hypothesis of fusobacteria’s hematogenous route seems plausible [18]. 

Regarding the abundance of *P. gingivalis* and the characteristics of GC in the studied groups, we found no correlation between them, while the scientific literature is inhomogeneous in this direction. *P. gingivalis* was found in high abundance in the saliva of patients with various digestive tract cancers, including GC, in comparison with the control group [19]. Some researchers discovered positive correlations between the levels of serum *P. gingivalis* IgG and mortality related to orodigestive cancers [44]. Higher levels of *P. gingivalis* were found in esophageal neoplastic tissues [45], and a recent review hypothesized about possible links between the bacterium and various digestive cancers, such as oral squamous cell carcinoma and esophageal, colorectal and pancreatic cancers [46]. Moreover, increased levels of *F. nucleatum* were found in the cancerous tissues of the esophagus when compared to the adjacent normal tissues and indicated a shorter cancer-specific survival, the presence of the bacterium being correlated with tumour stage [47]. Gallimidi et al. showed that in mice with tongue cancer which were repeatedly infected with *P. gingivalis* and *F. nucleatum*, tumour progression, growth and severity were upregulated because of the synergistic effect of the two bacteria [48]. Regarding esophageal squamous cell carcinoma and *P. gingivalis*, a recent study showed higher levels of the bacterium harvested from the oral biofilms in patients with esophageal cancer [49]. These inconsistent results motivated future studies to have a higher number of patients, to investigate the link between *P. gingivalis* and different characteristics of neoplastic tissues. 

A more complex oral microbiota in patients with GC was discovered, probably because of the weakened immunity which helps in creating a favourable environment for the bacteria, with increased levels of *Prevotella* and *Aggregatibacter* from the subgingival plaque and saliva in patients with GC and *Aggregatibacter* being found twice as often, compared with the control group [26]. 

The most abundant bacteria among the periopathogens investigated were *F. nucleatum* and *T. forsythia* in all groups, according to our findings. In more than half of the patients with GC from a study in Taiwan, strains of *Fusobacterium*, *Clostridium* and *Lactobacillus* were discovered, which could be used to identify GC with 100% sensitivity from endoscopic biopsies [12]. Cocker et al. [50] observed higher levels of *T. forsythia*, *Streptococcus anginosus* and *Peptostreptococcus stomatis* in stomach neoplastic tissues, in comparison to other precancerous lesions [50]. *F. nucleatum* and *C. colicanis* could be considered as diagnostic markers for early diagnosis of GC, along with biopsies through upper gastro-intestinal tract endoscopic examinations, which are considered an important method for the screening of patients [12,51]. The presence of these periopathogens in gastric tissues has not been linked with the periodontal status of patients or determined in the oral cavity, thus motivating the focus on these microorganisms in future larger studies in terms of the number of patients and their detection in neoplastic tissues.

Probably the most important feature of *F. nucleatum* is its ability to adhere to various host cells and bacteria [52]. *Fusobacterium* adhesin A (FadA) is one of the strongest virulence factors and is an important adhesion protein for the bacterium; thus, it can adhere to and invade epithelial and endothelial cells of the gingiva [52]. Our results revealed the presence of *FadA* in 13 of the 14 cases of the P group, all 12 cases of the SP group, but none of the cases of the G group, despite the fact that *F. nucleatum* was found in all cases of the G group, implying a possible role for *FadA*-positive strains of *F. nucleatum* in the periodontal pathology of GC patients. It was shown that the occurrence rate of *F. nucleatum* increases with the severity of periodontal lesions and that the *F. nucleatum* strain with *FadA* is thought to have increased pathogenicity, whereas the strain without *FadA* could represent a weak virulence genotype of the bacterium [53]. Another study revealed the involvement of *FadA*-positive strains of *F. nucleatum* in 33% of the tissue samples of patients with colorectal cancer [54], with the possible incriminating mechanisms being the *FadA*-mediated interaction with E-cadherin, thus highlighting increased tumour growth in xenograft mice [55]. Moreover, it was shown that the proliferation of cancer cells in stimulated by *FadA* [56].

Patients with colorectal cancer showed higher levels of *F. nucleatum* in tumour tissue, in comparison with healthy controls. Moreover, the bacterium was found in approximately half of the cases, which suggests a possible involvement in colorectal cancer, while some studies suggest a possible correlation with colorectal cancer metastasis [57,58]. Other authors hypothesized about possible links between increased levels of *F. nucleatum* and the recurrence of colorectal cancer and resistance to chemotherapy through the autophagy pathway [59].

The prevalence of poor differentiation grade in our study (slightly over 50%), in accordance with Feng et al. [60], was higher in patients with periodontitis, and it motivates the direction of future research on larger samples in order to investigate the possible correlation of DG with different periodontal parameters but also with the bacterial load of periopathogens in the gastric neoplastic tissue.

A potential limiting factor for the study could be the detection method used. For the purpose of this paper, we have used only target specific primers validated by other studies through random sample sequencing [53]. Although the primers used have sufficient discriminatory power, 16S rRNA-based PCR differentiations between highly phylogenetically related bacterial strains possess a risk of cross amplification, and in some cases, the desired accuracy is not achieved. More refined methods should be used to validate the findings of this study. 

Our study had a limited number of patients; thus, it could be stated that a broader study with a higher sample size in which the periopathogens are to be determined in the cancerous tissue as well is needed for a better understanding of the relationship between periodontal disease, periopathogens and gastric cancer. This present pilot study may motivate future research on a higher number of patients such that this relationship can be investigated further. 

## 4. Materials and Methods

### 4.1. Study Design

The current pilot study is a cross-sectional, transversal study, which was designed in accordance with the STROBE guidelines [61]. 

### 4.2. Setting

The study was performed between February and December 2020. The patients were recruited from the 1st Department of Surgery and Department of Gastroenterology of the Clinical County Hospital of Emergency of Craiova, Romania. The ethics approval for this study was obtained from the Research Ethic Commissions within the University of Medicine and Pharmacy of Craiova and Clinical County Hospital of Emergency of Craiova.

This study was carried out in full accordance with the World Medical Association Declaration of Helsinki, and all the patients included in this study signed an informed consent form.

### 4.3. Participants 

The study was performed on a sample of 24 patients with GC, aged 50–83 years, with a mean age of 69 ± 9.30. Thirteen were males (54.16%) with a mean age of 71.23 ± 9.71, and 11 (45.83%) were females with a mean age of 66.36 ± 8.47. The patients were hospitalized in the 1st Department of Surgery (16 patients) and the Department of Gastroenterology (8 patients) of the Clinical County Hospital of Emergency of Craiova. The participants were enrolled in this study if they had GC and could understand basic instructions. The criteria for exclusion were: patients unwilling or incapable of signing the informed consent form, unconsciousness, or if the oral examination could not be performed. 

Patients from the 1st Department of Surgery, previously diagnosed with GC, underwent radical surgery. Patients from the Department of Gastroenterology underwent an endoscopic examination during which a tumour biopsy was harvested for histological examination.

### 4.4. Periodontal Examination

The patients from the 1st Department of Surgery and Department of Gastroenterology diagnosed with GC underwent an oral clinical examination in the Dental Medicine Department by the same well-trained dentist (F.M.N.), recording the following variables: AT, PPD, CAL and BOP. All teeth were examined with a UNC15 periodontal probe (Hu-Friedy, Chicago, IL, USA) (except the 3rd molars and any remaining root tips) at 6 sites for each tooth (mesio-vestibular, centro-vestibular, disto-vestibular, mesio-lingual, centro-lingual and disto-lingual), concerning the immediate full millimetre. PPD and CAL were expressed in millimetres.

According to the 2018 classification of periodontal diseases and conditions [1,62], the patients with a minimum of 2 non-adjacent teeth present/a minimum of 5 teeth present were diagnosed with: gingivitis if they had BOP greater than/equal to 10% without CAL and periodontitis if they presented interdental CAL at 2 non-adjacent teeth/ more or vestibular/oral CAL greater than/equal to 3 mm with PPD greater than/equal to 3 mm in 2 teeth/more. The patients who did not meet the previous criteria were considered periodontally healthy.

After the clinical examination, all 24 patients showed the periodontal modifications mentioned above and were matched as follows: P group—14 subjects (5 women, 9 men, 12 from Surgery Department, 2 from Gastroenterology); G group—10 subjects (6 women, 4 men, 4 from Surgery Department and 6 from Gastroenterology). For the patients from the Surgical Department, TD could also be determined, as a characteristic of gastric cancerous samples, in comparison with the patients from the Gastroenterology Department, where only DG could be determined (Section 4.7).

### 4.5. Gingival Crevicular Fluid Sampling

After the periodontal clinical examination, for the P group, the gingival crevicular fluid was harvested from the tooth with the deepest periodontal pockets. For the G group, the samples were collected from the tooth with the most severe BOP. Each tooth was isolated with cotton rolls and then air-dried. With another cotton roll, the supragingival plaque was removed. Gingival crevicular fluid samples were collected by the intracrevicular method, through the absorbing technique, using a sterile paper cone, size 60 (Dentsply-De-Trey^®^, Ballaigues, Switzerland). The paper cone was inserted in the gingival sulcus for 30 s and then placed in cryo-tubes of 2 mL with 500 µL of RNA Save^®^ solution (Biological Industries, Haemek, Israel). The samples were stored at −80 °C, and they were preserved until their use for DNA extraction. 

### 4.6. PCR Assessment

#### 4.6.1. DNA Extraction

DNA extraction was performed using the commercial extraction kit PureLink^®^ Genomic DNA Mini Kit (Invitrogen, Waltham, MA, USA). Simultaneous with the extraction protocol, we carried out the contamination control between samples, by processing a sample of water with a purity for molecular biology.

The quantification and the purity of the isolated DNA were examined through UV/Vis nanospectrophotometry. We used a NanoPhotometer^®^ (ImplenGmbh, München, Germania). The quantification was determined at the optic density (OD) of 260 nm, and the ratio OD_260 nm_/OD_280 nm_ indicated the purity of DNA. Values of the ratio OD_260 nm_/OD_280 nm_ of 1.7–2.0 signified pure DNA. 

#### 4.6.2. Quantification of Periopathogens

The sequence of the primers and the probes used for the quantitative analysis of periopathogens through qPCR is described in Table 3. The primers aimed for the high-conserved regions of the genes *waaA* (kdtA) and *waaG*, with the exception of *F. nucleatum* [53,63]. 

The same genes can be found in the genome of the species listed above in a single copy, which simplifies the quantification of the bacteria. The qPCR result represents the number of amplicons, and in this case, because the amplicon follows a fragment of a gene in a single copy, it translates directly into the number of copies in the genome, equivalent to the number of bacteria. 

For the construction of the standard curve and as a positive control, a recombinant plasmid, pUC57, was used, in which the DNA sequence of interest was flanked by sequences complementary to the primers and which was incorporated to allow for their attachment. This synthetic plasmid was purchased from Eurogentec^®^, Belgium. Decimal dilutions of the positive control 10–10^7^ copies/reaction were performed. Bio pure water was used as a negative control to replace the DNA isolated from the test sample. 

Periopathogens quantification was performed by real-time quantitative PCR, TaqMan method, using the Mx3005P qPCR platform (Stratagene^®^, San Diego, CA, USA). The qPCR reactions were performed in a total volume of 25 µL of which 2 µL of the DNA isolated from the sample was to be analysed. Then, 12.5 µL of GoTaq^®^ Probe qPCR Master Mix solution, 0.4 µL ROX, and the volumes of primer, probe and bio pure water were properly optimized to determine effective concentration. In the case of *A. actinomycetemcomitans*, the concentrations of primers and probes were 100 and 200 nM, respectively, for *P. gingivalis*, 300 and 200 nM, for *T. denticola*, 300 and 100 nM, and in the case of *T. forsythia*, 100 and 100 nM. The amplification was performed after the following thermal program: initial denaturation at 95 °C for 10 min and 40 cycles of 95 °C for 30 s, 60 °C for 1 min [63].

Detection and quantification of *F. nucleatum* 16S rRNA and *fadA* genes were performed by qPCR using intercalated fluorochromes that attach specifically to double-stranded DNA. In the case of the 16S rRNA gene, the primers targeted and amplified the 408 bp theoretical fragment between position 43 and 450 of the 16S rRNA gene of the species *F. nucleatum* subsp. nucleatum ATCC 25586, while the primers for *fadA* amplified a 232 bp fragment between position 155 and 386 of the *fadA* gene [53].

The same quantification strategy was followed with synthetic plasmid as in the case of periopathogens from the Socransky’s red complex. The qPCR reactions were performed in a total volume of 25 µL, of which 2 µL of DNA isolated from the test sample, 12.5 µL of GoTaq^®^ qPCR Master Mix solution, 0.4 µL ROX, primer in a final concentration of 300 nM, and bio pure water. The following thermocycling profile was used: initial denaturation at 95 °C for 10 min and 40 cycles of 95 °C for 30 s, 58 °C for 1 min [53]. 

### 4.7. Histological Analysis of the Gastric Tissue

The GC tissue samples obtained from endoscopic biopsies or a gastrectomy were examined by the same pathologist using the standard histopathological technique, and the diagnostic was adenocarcinoma. A 10% formalin solution was used to fix the harvested tissue material. The fragments taken in the process of macroscopic orientation of the piece were processed in the classical histological technique of inclusion in paraffin, progressing through the following phases: dehydration, clarification, paraffining, actual inclusion, sectioning and staining. For the basic histopathological examination, the haematoxylin-eosin (HE) staining technique was used.

On the histopathological exam, for all the gastric biopsies, the DG was recorded as: well differentiated in comparison with healthy adjacent tissues, moderately differentiated, and poorly differentiated compared to healthy adjacent tissues [60]; for the gastrectomy samples from patients from the Surgery Department, tumour size (TD) was also taken into consideration. TD was expressed in millimetres and concerned the maximum diameter of the neoplastic tissue.

### 4.8. Statistical Analyses

Data expressed as mean and standard deviation (SD) were subjected to statistical analysis SPSS 19.0 (IBM, Chicago, IL, USA) in order to detect the differences between groups, using the Mann–Whitney test (statistically significant < 0.05%), due to the small number of patients. The existence of statistical correlations between the different types of data using Pearson’s and Spearman’s coefficients was assessed. The analysis was performed after a power computation (G*Power 3, University of Dusseldorf, Dusseldorf, Germany) that revealed that, for the G, P and SP groups, the power for different analyses was between 88% and 95%.

## 5. Conclusions

Within the limitations of this study, the results suggest an association between periodontal disease, the subgingival periopathogens, especially *F. nucleatum*, and gastric cancer. 

## Figures and Tables

**Table 1:** Presence of bacteria in the gingival crevicular fluid of gastric cancer patients and the periodontal parameters.

Periodontal Status	*F. nucleatum*	*P. gingivalis*	*T. denticola*	*T. forsythia*	*A. actinomycetemcomitans*	fadA	AT	PPD	CAL	BOP
	% (*n*)						Mean ± SD			
G	100 (*10*)	60 (*6*)	70 (*7*)	90 (*9*)	0 (*0*)	0 (*0*)	5.9 ± 1.72	1.37 ± 0.24	0	14.22 ± 2.22
P	100 (*14*)	85.71 (*12*)	78.57 (*11*)	100 (*14*)	35.71 (*5*)	92.85 (*13*)	9.35 ± 2.92	6.27 ± 0.75	4.02 ± 0.74	47.49 ± 12.23

*F. nucleatum*, *Fusobacterium nucleatum*; *P. gingivalis*, *Porphyromonas gingivalis*; *T. denticola*, *Treponema denticola*; *T. forsythia*, *Tannerella forsythia*; *A. actinomycetemcomitans*, *Aggregatibacter actinomycetemcomitans*; *fadA*, FadA adhesin; %, percentage of patients from the group; *n*, number of patients; AT, number of absent teeth; PPD, probing pocket depth; CAL, clinical attachment loss; BOP, bleeding on probing; SD, standard deviation; G, gingivitis group; P, periodontitis group.

**Table 2:** DG in the three studied groups.

	G	P
DG	well/moderate/poor	well/moderate/poor
%	20/30/50	14.28/28.57/57.14

DG, differentiation grade; G, gingivitis group; P, periodontitis group; %, percentage of patients in each group.

**Table 3:** Sequence of primers and probes.

Pathogen *	Primer 5′→3′	Probe 5′→3′	Gene
*A.* *actinomycetemcomitans*	F: GCGAACGTTAGCGTTTTAC R: GGCAAATAAACGTGGGTGAC	AATTGCCCGCACCGAAACCCAAC 5′_Cy5→BHQ2_3′	*waaA*
*P. gingivalis*	F: TGGTTTCATGCAGCTTCTT R: TCGGCACCTTCGTAATTCTT	CGTACCTCATATCCCGAGGGGCTG 5′_HEX→BHQ1_3′	*waaA*
*T. denticola*	F: CCTTGAACAAAAACCGGAA R: GGGAAAAGCAGGAAGCATAA	GAGCTCTGAATAATTTTGATGCA 5′_Cy5→BHQ2_3′	*waaG*
*T. forsythia*	F: CTCGCTCGGTGAGTTTGAA R: ATGGCGAAAAGAACGTCAAC	CGATTCGCAAGCGTTATCCCGACT 5′_HEX→BHQ1_3′	*waaA*
*F. nucleatum*	F: AGAGTTTGATCCTGGCTCAG R: GTCATCGTGCACACAGAATTGCTG		16S rRNA
*fadA*	F: CACAAGCTGACGCTGCTAGA R: TTACCAGCTCTTAAAGCTTG		*fadA*

Pathogens *: *A. actinomycetemcomitans*, *Aggregatibacter actinomycetemcomitans*; *P. gingivalis*, *Porphyromonas gingivalis*; *T. denticola*, *Treponema denticola*; *T. forsythia*, *Tannerella forsythia*; *F. nucleatum*, *Fusobacterium nucleatum*; *fadA*, FadA adhesin.

## Data Availability

The data used to support the findings of this study are available from the corresponding author upon reasonable request.

## Abbreviations

AT	Absent teeth
BOP	Bleeding on probing
CAL	Clinical attachment loss
DG	Differentiation grade
G	Gingivitis group
GC	Gastric cancer
*p*	Statistical significance
P	Periodontitis group
PD	Periodontal disease
PPD	Probing pocket depth
r	Correlation grade
SD	Standard deviation
SG	Patients from 1st Department of Surgery with gingivitis
SP	Patients from 1st Department of Surgery with periodontitis
TD	Tumour dimension
